# Research on Detection Performance of NaI(Tl) Detector Based on Monte Carlo Method

**DOI:** 10.3390/s26123913

**Published:** 2026-06-19

**Authors:** Qingbo Du, Yapeng Yang, Xiaoyu Zhao, Qi Lv, Yuyao Tang, Jiapeng He, Yier Liu, Guoqiang Li

**Affiliations:** 1 China Institute for Radiation Protection, Taiyuan 030006, China; duqingbo@cirp.org.cn (Q.D.); yangyapeng@cirp.org.cn (Y.Y.); zhaoxiaoyu@cirp.org.cn (X.Z.); lvqi@cirp.org.cn (Q.L.); tangyuyao@cirp.org.cn (Y.T.); hejiapeng@cirp.org.cn (J.H.); liuyear65@gmail.com (Y.L.); 2 Shanxi Provincial Key Laboratory of Nuclear and Radiation Emergency Response, Taiyuan 030006, China

**Keywords:** Monte Carlo method, NaI(Tl) detector, detection performance, detection efficiency, energy resolution, peak-to-total ratio

## Abstract

The NaI(TI) detector is highly favored in gamma radiation detection and widely applied in fields such as environmental radiation monitoring, nuclear medicine, and laboratory gamma-ray spectroscopy. Its detection performance determines the results of quantitative gamma-ray detection, making it a crucial indicator in detector design and development. This study employs the Monte Carlo method and utilizes TopMC 1.0 software to establish a NaI(TI) detector model. First, the effects of crystal size, ray energy, cladding thickness, and distance on the detector’s detection efficiency were investigated. Subsequently, the energy resolution and peak-to-total ratio of the detector were simulated and calculated, with comparisons made to experimental values. The results indicate that all three detection efficiencies of the NaI(TI) detector are positively correlated with crystal size and exhibit an initial increase followed by a decrease with rising gamma-ray energy. Both the absolute detection efficiency and full-energy peak detection efficiency first increase and then decrease with increasing cladding thickness, while showing a negative correlation with detection distance. The intrinsic detection efficiency is almost unaffected by cladding thickness and initially rises before declining with increasing detection distance. The simulated values of energy resolution closely match experimental values, improving with higher gamma-ray energy. The deviation between simulated and experimental values for different source peak-to-total ratios remains within 6.25%, verifying the model’s reliability and the accuracy of simulation data. These findings provide valuable references and guidance for optimizing detection performance, conducting source-free efficiency calibration, and structural design of NaI(TI) detectors.

## 1. Introduction

The development of nuclear instrumentation and radiation detection methods relies on modern technologies derived from fundamental research. Scintillator materials are widely used in nuclear physics and extensively employed in radiation detection. NaI(Tl) crystals are the most commonly used radiation detection material for scintillator-based nuclear radiation detectors. With their high density, high atomic number, excellent performance in detecting gamma rays and other ionizing radiation, and availability in various sizes, NaI(Tl) detectors developed based on this material offer high detection efficiency, low power consumption, and affordable prices, making them highly favored in gamma radiation detection. In the structural design and performance optimization of detectors, key structural parameters such as the geometric dimensions of the detection crystal and the thickness of the encapsulation shell determine the core detection performance metrics, making them essential aspects for optimization research in the engineering design of nuclear radiation detectors. During radiation detection, since detector parameters like detection efficiency and energy resolution are crucial for accurate γ-ray detection and quantitative analysis, it is necessary to precisely understand the relevant detection performance of the detector. Typically, the detector’s performance is calibrated through radiation detection experiments. However, due to limited experimental facilities, difficulties in obtaining standard sources, the restricted number of available ray energies, high costs, and the lack of diverse radioactive sources, it is challenging to achieve the direct detection of γ-rays at multiple distances and different energies. The Monte Carlo (MC) method is frequently applied in the study of nuclear radiation detector performance, due to its capability to simulate the transport process of radiation particles within the detector structure under experimental conditions. Previous studies have employed the Monte Carlo method to investigate NaI(Tl) detectors. Cengiz et al. [1] (2008) used Monte Carlo simulations to study the response function of a 3 × 3 inch NaI(Tl) detector to γ-rays from a point source located 10 cm away. Hajheidari et al. [2] (2016) utilized MCNPX and FLUKA Monte Carlo software to simulate the effectiveness of the NaI(Tl) detector’s response function, showing that the deviation between the simulated and experimental values was less than 8%. In the same year, Ahmed et al. [3] simulated the absolute and intrinsic detection efficiency of a cylindrical NaI(Tl) detector using the Monte Carlo method, with their results indicating a deviation of less than 2% between the simulated and experimental data. Mouhti et al. [4] (2017) evaluated the absolute detection efficiency of spherical and cylindrical NaI(Tl) detectors using MCNP4C, demonstrating that the software could successfully simulate the detector’s response function. Also in 2017, Olaniyi et al. [5] simulated the response function of a 3 × 3 inch NaI(Tl) detector based on the Monte Carlo method, reporting deviations of 0.01% and 0.66% between the simulated and experimental energy resolutions for ^137^Cs and ^60^Co, respectively. Demir et al. [6] (2021) employed the FLUKA Monte Carlo software to study the energy resolution of NaI(Tl) detectors, showing consistency between the simulated, experimental, and theoretical values, which could provide accurate pre-calculation for detector design. In the same year, Grozdanov et al. [7] used Geant4 to model the crystal and studied the response function of NaI(Tl) detectors at 662 keV, demonstrating that the variation in efficiency with energy agreed with the experimental results to within 10%. Tekin et al. [8] (2022) modeled a standard NaI(Tl) detector using MCNPX, proving it to be an effective instrument for obtaining fundamental γ-ray parameters such as mass attenuation coefficients. Wang et al. [9] (2023) conducted a simulation based on the Monte Carlo method for a NaI(Tl) detector with dimensions of Φ7.5 cm × 7.5 cm, obtained gamma-ray energy spectra in the energy range of 50 keV to 2.5 MeV, and studied its dose rate detection performance; the results indicate that the dose rate values calculated by the simulation have a small deviation from the experimental values. Nahid et al. [10] (2024) investigated the photopeak efficiency response function and total efficiency of NaI(Tl) detectors using MCNPX, with the simulations showing good agreement with the experiments. Fatima et al. [11] (2025) modeled NaI(Tl) detectors using Geant4 and calculated the absolute efficiency, intrinsic efficiency, and mass attenuation coefficients for three different absorbers (Pb, Al, and Cu) at various energies and thicknesses, with the results consistent with the experimental data, validating the reliability of Monte Carlo methods and software for precise modeling and performance studies of NaI(Tl) detectors.

Based on the Monte Carlo method, this paper conducts a comparative study on the γ-ray detection efficiency of cylindrical NaI(Tl) crystals with different dimensions (Φ1 inch × 1 inch indicates that both the diameter and height are 1 inch, Φ2 inch × 2 inch, Φ3 inch × 3 inch). The comparison results indicate that the Φ3 inch × 3 inch NaI(Tl) crystal exhibits the best radiation detection efficiency. Using the Φ3 inch × 3 inch NaI(Tl) crystal as the basis, this paper thoroughly investigates the effects of incident γ-ray energy, thickness of the encapsulation material, and distance between the radiation source and detector on the detection efficiency of the NaI(Tl) detector. Additionally, studies are performed on its energy resolution and peak-to-total ratio. In this paper, an innovative attempt was made to conduct a multi-dimensional and systematic study on the factors affecting the detection performance of NaI(Tl) detectors using the domestic TopMC software. The physical mechanism of how these factors influence the detector’s detection performance was revealed, and research conclusions were distilled. The research findings can provide valuable references for scintillation detector structural design, product development, and passive efficiency calibration.

## 2. Theoretical Foundation and Model Establishment

### 2.1. Theoretical Foundation

#### 2.1.1. Detection Efficiency

The detection efficiency represents the relationship between the radiation pulse counts detected by the detector and the activity of the radiation source. It depends on factors such as the type and size of the detection material, as well as the geometric structure of the detector. When detecting gamma rays, based on different incident gamma photon forms (such as different incident energies, incident directions, and incident beam configurations, etc.) and various pulse count statistical methods in gamma spectrometers, the detection efficiency can be classified into three types: absolute detection efficiency, full-energy peak detection efficiency, and intrinsic detection efficiency.

The absolute detection efficiency is defined as the ratio of the total pulse count in the detector’s gamma spectrum to the total number of particles emitted by the radiation source during the detection time, with the calculation formula as follows:

(1) $$\varepsilon_{abe}=\frac{N}{N_{0}}.$$

In the equation, *ε_abe_* represents the absolute detection efficiency, *N* is the total count of the detector’s gamma spectrum, and *N_0_* is the total number of particles emitted by the radiation source during the detection period.

The full-energy peak detection efficiency is defined as the ratio of the counts in the full-energy peak of the detector’s gamma spectrum to the total number of particles emitted by the radiation source during the same time period. The calculation formula is as follows:

(2) $$\varepsilon_{p}=\frac{N_{peak}}{N_{0}}.$$

In the equation, *ε_p_* is the full-energy peak detection efficiency, and *N_peak_* is the full-energy peak count in the gamma spectrum of the detector.

The intrinsic detection efficiency is defined as the ratio of the total pulse count in the detector’s gamma spectrum to the number of particles incident on the sensitive volume of the detector, with the calculation formula as follows:

(3) $$\varepsilon_{in}=\frac{N}{N_{i}}.$$

In the equation, *ε_in_* is the intrinsic detection efficiency, and *N_i_* is the number of particles incident on the sensitive volume of the detector.

#### 2.1.2. Energy Resolution

Energy resolution is a core performance indicator characterizing a detector’s ability to distinguish differences in incident radiation energy. A smaller value indicates stronger energy discrimination capability. It is defined as the ratio of the full width at half maximum (*FWHM*) of the full-energy peak in the gamma spectrum measured by the detector to the corresponding peak energy, typically expressed as a percentage. The calculation formula is as follows:

(4) $$R=\frac{FWHM}{E}\times 100\%.$$

In the equation, *R* represents the energy resolution of the detector, whose theoretical limit is determined by intrinsic statistical fluctuations, and *E* denotes the energy of the incident monoenergetic radiation (the energy corresponding to the full-energy peak).

#### 2.1.3. Peak-to-Total Ratio

The peak-to-total ratio represents the probability of γ-ray particles entering the sensitive volume of the detector producing a full-energy peak, which is the ratio of full-energy peak counts to total counts in the detector’s γ-ray spectrum. The calculation formula is as follows:

(5) $$\varepsilon_{f}=\frac{N_{peak}}{N}=\frac{\varepsilon_{p}}{\varepsilon_{abe}}$$

In the equation, *ε_f_* is the peak-to-total ratio, which is commonly used to directly measure the detection efficiency of the full-energy peak produced by the detector after absorbing γ-rays.

### 2.2. Monte Carlo Model Establishment

**Figure 1:**
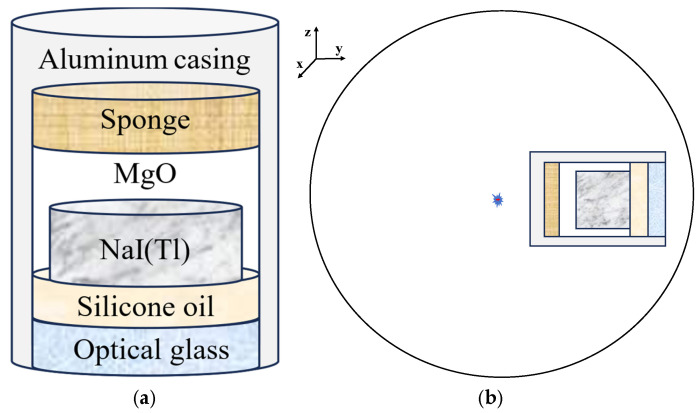
Structure diagram of NaI(Tl) scintillation detector: (**a**) model structure diagram of NaI(Tl) scintillation detector; (**b**) schematic diagram of MC simulation geometry.

Referencing the actual structure of a NaI(Tl) detector, this study established a simulation model of a cylindrical NaI(Tl) scintillation detector in the TopMC software [12]. The model structure diagram and the Monte Carlo simulation geometry schematic are shown in Figure 1.

The NaI(Tl) scintillation detector is primarily encapsulated with a sponge, MgO reflective layer, NaI(Tl) crystal, silicone oil, optical glass, and an aluminum housing. The specific structure consists of 0.1 cm aluminum housing, 0.1 cm sponge, and a 0.1 cm MgO layer sequentially above the NaI(Tl) crystal; 0.1 cm MgO and 0.1 cm aluminum housing on the sides; and 0.1 cm silicone oil with 0.2 cm optical glass beneath. In this study, the simulated particle count was set to 1 × 10^7^ to ensure computational efficiency while maintaining result accuracy.

## 3. Analysis of Simulation Calculation Results

### 3.1. The Effect of Crystal Size on Detection Efficiency

When studying the influence of the NaI(Tl) crystal size on the detector detection efficiency, this research set the simulated radiation source as a ^137^Cs isotropic point source (γ-ray energy 0.662 MeV), with the distance between the point source and the detector fixed at 5 cm (the distance from the source to the nearest surface of the detector). Using the Monte Carlo method, the energy response of the NaI(Tl) detector was simulated for different crystal sizes (Φ1 inch × 1 inch, Φ2 inch × 2 inch, Φ3 inch × 3 inch), and the detector energy spectrum data were obtained. Through Equations (1)–(3), the absolute detection efficiency, full-energy peak detection efficiency, and intrinsic detection efficiency corresponding to different crystal sizes were calculated, as shown in Table 1.

As can be seen from Table 1, for detectors with the same crystal size, the absolute detection efficiency, full-energy peak efficiency, and intrinsic detection efficiency follow the same trend: *ε_in_* > *ε_abe_* > *ε_p_*. This is because *ε_in_* only accounts for the probability of particles interacting within the sensitive volume of the detector, without constraints from spatial transport or solid angle losses, resulting in the highest value. *ε_abe_* is calculated based on particles emitted isotropically from the source, incorporating those that fail to reach the sensitive volume due to geometric solid angle limitations and transport attenuation, leading to a lower value. *ε_p_* exclusively counts events with complete energy deposition, excluding cases like the Compton escape where only partial energy is deposited, thus yielding the lowest value. As the crystal size increases, all three detection efficiencies continuously improve. This is primarily because the larger crystal size increases the effective interaction path of radiation, the sensitive detection volume, and the solid angle of the detector relative to the radiation source. These enhancements expand the interaction range between radiation particles and the crystal, increase the probability of energy deposition, and reduce the probability of incident radiation particles penetrating the crystal without interaction (leakage counting). Consequently, the energy deposition efficiency of radiation and the proportion of detectable events are significantly improved, thereby enhancing the detection efficiency. In subsequent studies, the NaI(Tl) crystal was set to the size with the best detection efficiency performance (i.e., Φ3 inch × 3 inch), and the reflector specification was optimally selected as 0.1 cm thick MgO [13].

### 3.2. The Effect of Ray Energy on Detection Efficiency

**Figure 2:**
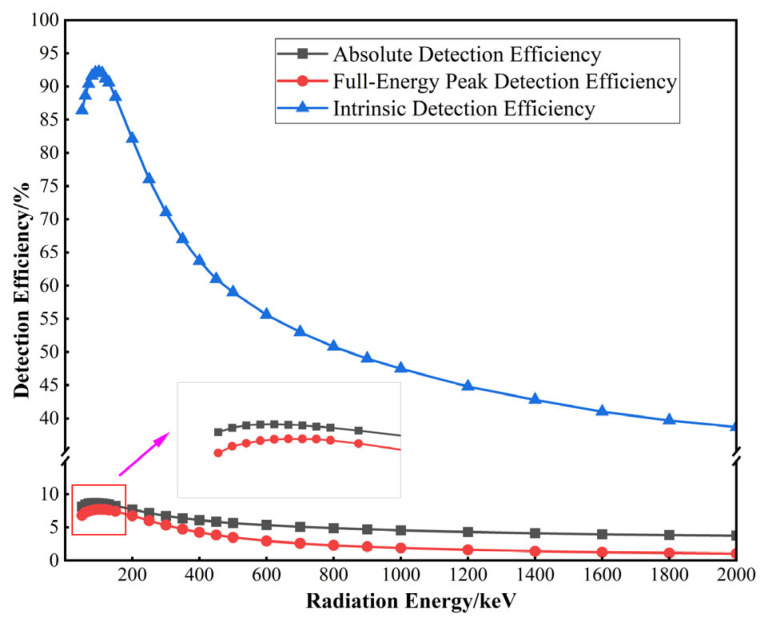
The variation trend in detection efficiency with γ-ray energy.

When studying the influence of γ-ray energy on the detection efficiency, the energy range was set from 50 to 2000 keV. Six data groups were collected with 10 keV intervals between 50 and 100 keV, eight groups with 50 keV intervals between 100 and 500 keV, five groups with 100 keV intervals between 500 and 1000 keV, and five groups with 200 keV intervals between 1000 and 2000 keV. The distance between the point source and detector was set at 5 cm, with the simulated radiation source configured as an isotropic point source. The research results are shown in Figure 2.

As can be seen from Figure 2, all three detection efficiencies exhibit an initial increase followed by a decrease with increasing γ-ray energy. The absolute detection efficiency, full-energy peak detection efficiency, and intrinsic detection efficiency all reach their maximum values at a γ-ray energy of 100 keV, measuring 8.63%, 7.72%, and 92.2% respectively. Furthermore, for both the increasing and decreasing trends, the intrinsic detection efficiency shows a higher variation amplitude compared to the absolute detection efficiency and full-energy peak detection efficiency. When the γ-ray energy exceeds 1400 keV, the decline in all three detection efficiencies tends to stabilize. The main reason is that when the γ-ray energy is below 100 keV, as the γ-ray energy increases, the reduction in particle transport attenuation (meaning more particles reach the sensitive volume of the detector) becomes the dominant factor, leading to a significant improvement in intrinsic detection efficiency compared to the other two detection efficiencies. When the γ-ray energy exceeds 100 keV, as the γ-ray energy increases, the increasing probability of leakage counts (where particles pass through the sensitive volume of the detector without interaction or energy deposition) becomes the dominant factor, causing a more pronounced decline in intrinsic detection efficiency. Additionally, the proportion of Compton effects gradually increases, resulting in a more noticeable downward trend in full-energy peak detection efficiency compared to absolute detection efficiency. Therefore, NaI(Tl) detectors yield better results when used for detecting low-energy γ radiation.

### 3.3. The Effect of Cladding Thickness on Detection Efficiency

**Figure 3:**
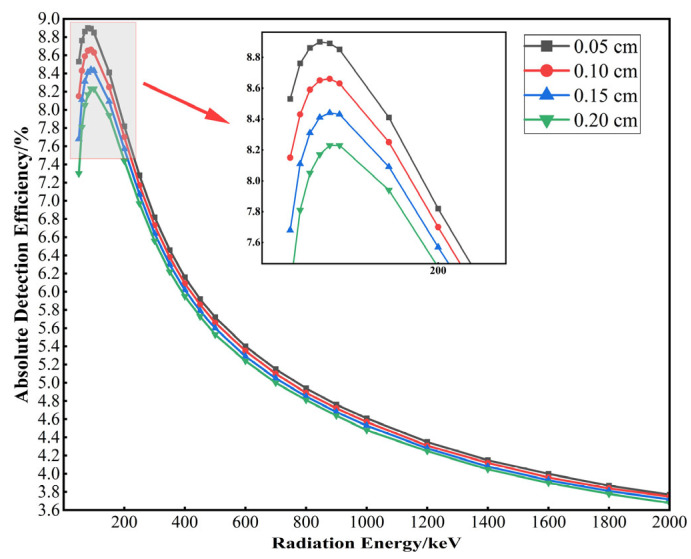
The curve of absolute detection efficiency vs. γ-ray energy under aluminum shells of different thicknesses.

**Figure 4:**
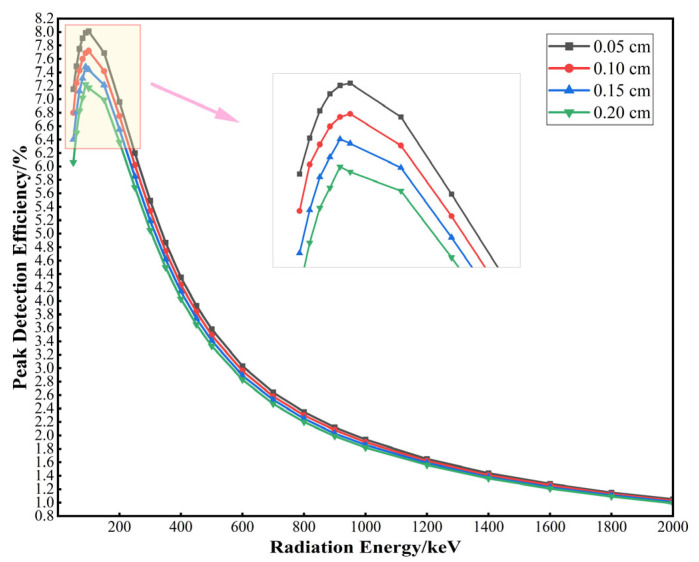
The variation curve of full-energy peak detection efficiency with γ-ray energy under aluminum shells of different thicknesses.

Aluminum is a commonly used cladding material for detectors, with the advantage of being relatively sturdy, but it has the drawback of strong absorption capability for low-energy γ-rays and X-rays. When studying the influence of the thickness of the detector entrance window cladding material on detection efficiency, the distance between the radiation source and the detector incident window was set to 5 cm. The incident γ-ray energy was consistent with that used in the study of the effect of radiation energy on detection efficiency. Four aluminum shell incident windows with thicknesses of 0.05 cm, 0.1 cm, 0.15 cm, and 0.2 cm were selected for simulation, and the trend curves of three types of detection efficiencies under different aluminum shell thicknesses as a function of γ-ray energy were obtained. Figure 3 and Figure 4 show the absolute detection efficiency and full-energy peak detection efficiency of the detector under fthe our different aluminum shell thicknesses as a function of incident γ-ray energy, respectively.

As can be seen from Figure 3 and Figure 4, under aluminum shell incident windows with thicknesses of 0.05 cm, 0.1 cm, 0.15 cm, and 0.2 cm, the absolute detection efficiency and full-energy peak detection efficiency of the detector exhibit consistent trends with varying γ-ray energy, both initially increasing and then decreasing, reaching their maximum values at approximately 100 keV γ-ray energy. Across the simulated γ-ray energy range (50–2000 keV), both the absolute detection efficiency and full-energy peak detection efficiency decrease, as the aluminum shell thickness increases. This is attributed to the enhanced stopping power of the aluminum shell against γ-rays with increasing thickness, which reduces the number of incident radiation particles reaching the sensitive volume of the detector, consequently leading to decreased absolute detection efficiency and full-energy peak detection efficiency.

**Figure 5:**
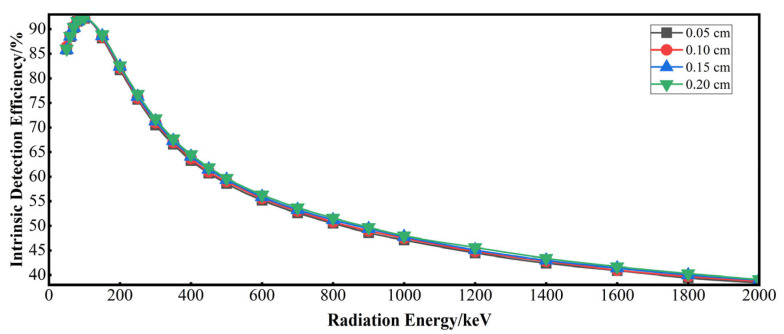
The intrinsic detection efficiency vs. γ-ray energy curves for aluminum shells of different thicknesses.

Figure 5 shows the variation trend of the intrinsic detection efficiency of the detector with different aluminum shell thicknesses as a function of the incident gamma-ray energy.

As can be seen in Figure 5, under different aluminum shell thicknesses, the intrinsic detection efficiency of the detector first increases and then decreases, as the γ-ray energy increases, reaching its maximum at approximately 100 keV. The aluminum shell thickness has almost no effect on the intrinsic detection efficiency of the detector. Therefore, to achieve the optimal detection efficiency, the aluminum shell thickness on the detector incident window should be minimized while ensuring the mechanical structural strength of the detector.

### 3.4. The Effect of Distance on Detection Efficiency

**Figure 6:**
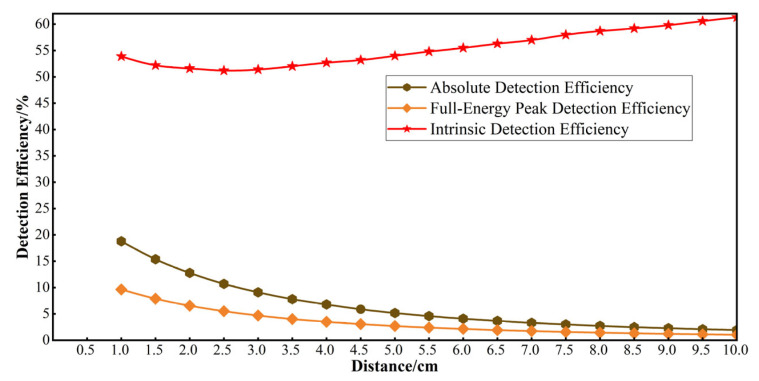
Detection efficiency vs. distance trend curve.

When studying the influence of the distance between the radiation source and the detector on the detection efficiency, the radiation source was configured as a ^137^Cs isotropic point source with a standard 0.1 cm thick aluminum front shell. The distance between the radiation source and the detector was varied from 1 cm to 10 cm in 0.5 cm increments, resulting in 19 data sets. Figure 6 shows the three detection efficiency curves as functions of distance obtained from this study.

As can be seen from Figure 6, both the absolute detection efficiency and the full-energy peak detection efficiency decrease, as the distance between the radiation source and the detector increases, with roughly similar declining trends. This occurs because the solid angle subtended by the detector for the radiation source continuously decreases with increasing distance, causing the radiation to become more divergent. Consequently, the number of rays reaching the detector’s front end decreases, leading to a reduction in both absolute detection efficiency and full-energy peak detection efficiency. Therefore, an excessively large detection distance results in unacceptably low detection accuracy. In contrast, the intrinsic detection efficiency first decreases and then increases with the detection distance, reaching its minimum value of 51.2% at a detection distance of 2.5 cm. The physical origin of this non-monotonic variation lies in the fact that the distribution of the incident angle of the gamma-ray beam (referring to the angle between the incident ray and the crystal axis direction) changes with detection distance, causing the average effective path of the incident gamma-ray beam within the detection crystal to vary non-monotonically. When the detection distance is small, the range of incident angles of the gamma-ray beam is wide, and the effective path of a large number of incident rays through the crystal exceeds the crystal thickness, resulting in a higher interaction probability. As the detection distance increases, the proportion of incident rays with large incident angles decreases, the average effective path shortens, and the intrinsic detection efficiency decreases. When the two competing effects—the attenuation of the proportion of large-angle incident rays and the approach of the path of small-angle incident rays to the detection crystal thickness—reach a balance, the average effective path of the incident ray beam reaches a minimum, and the intrinsic detection efficiency drops to a minimum. Thereafter, as the detection distance continues to increase, the incident gamma-ray beam gradually becomes more parallel, and the average effective path slowly increases, leading to a slow increase in intrinsic detection efficiency.

### 3.5. Energy Resolution

**Figure 7:**
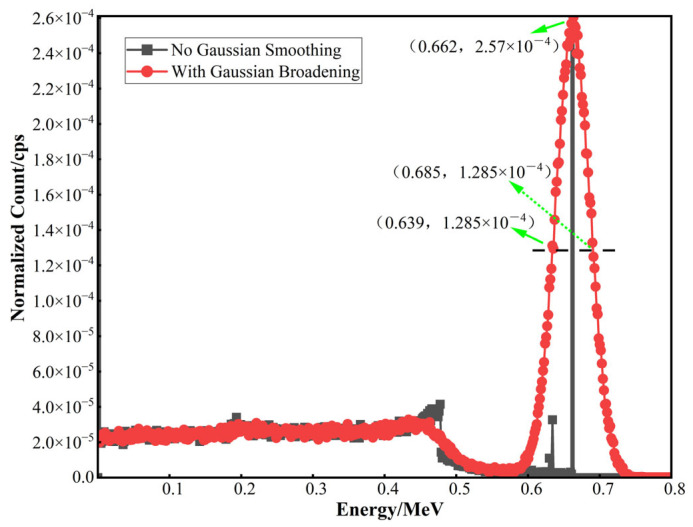
Simulated spectrum of ^137^Cs radiation.

When studying the energy resolution of the detector, the simulated radiation source was set as a ^137^Cs isotropic point source, with a distance of 10 cm between the source and the detector. The thickness of the aluminum shell at the front end of the detector was 0.1 cm, and the number of simulated particles was 1 × 10^7^. The energy deposition spectrum of γ-rays obtained from the simulation could not reflect the influence of statistical fluctuations of rays and the detector on the actual energy spectrum during the detection process. The characteristic peaks in the simulated energy deposition spectrum also did not exhibit a Gaussian distribution, making it impossible to calculate the energy resolution of the detector. Therefore, the Gaussian broadening function was applied to the pulse amplitude distribution of the spectrum to perform Gaussian broadening [14,15], resulting in the ^137^Cs energy spectrum shown in Figure 7.

As shown in Figure 7, the energy corresponding to the full-energy peak in the spectrum matches the γ-ray energy emitted by the simulated ^137^Cs source. The full width at half maximum (*FWHM*) of the peak is 0.046 MeV. Substituting this value into the energy resolution calculation Formula (4), the energy resolution of the NaI(Tl) detector is approximately 6.95%@^137^Cs (662 keV). This result is consistent with the optimal energy resolution value of 7% for 662 keV γ-rays emitted by ^137^Cs, as reported in the literature [10] for a Φ3-inch × 3-inch NaI(Tl) detector, thereby verifying the reliability of the detector model.

**Figure 8:**
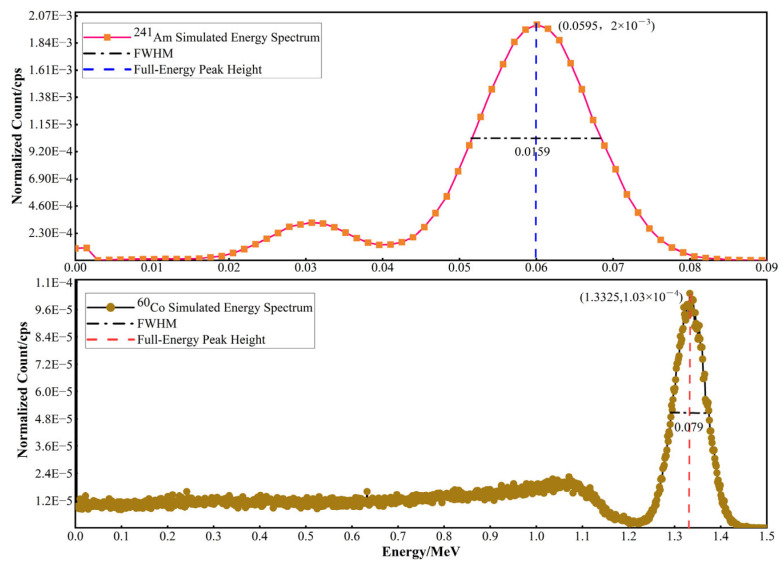
Simulated energy spectra of ^241^Am and ^60^Co.

To further investigate the energy resolution of the detector for γ-rays of different energies, the simulated radiation sources were set as isotropic point sources of ^241^Am with an energy of 59.5 keV and ^60^Co with an energy of 1332.5 keV, respectively, resulting in the energy spectrum shown in Figure 8.

As can be seen from Figure 8, the energies at the full-energy peaks in the simulated spectra of ^241^Am and ^60^Co are consistent with the γ-ray energies emitted by the sources. The calculated full width at half maximum (*FWHM*) and energy resolution values for each simulated source spectrum are shown in Table 2.

As can be seen from Table 2, the simulated energy resolution values of the detector for γ-rays emitted from ^137^Cs and ^60^Co sources are close to the experimental values, while the simulated energy resolution for γ-rays from the ^241^Am source shows a relatively more significant deviation from the experimental value. Moreover, as the energy of the γ-rays increases, the simulated energy resolution values of the detector decrease, which is consistent with the trend observed in the experimental values. This is primarily because the relative contribution of statistical fluctuations affecting the energy resolution significantly decreases within this energy range as the incident γ-ray energy increases, leading to poorer energy resolution at the low-energy γ-rays of the ^241^Am source and improved energy resolution with increasing γ-ray energy.

### 3.6. Peak-to-Total Ratio

When studying the peak-to-total ratio of the detector, the simulated radiation sources were set as 10 isotropic point sources including ^47^Sc, ^139^Ce, ^51^Cr, ^7^Be, ^137^Cs, ^95^Nb, ^54^Mn, ^65^Zn, ^28^Al, and ^88^Y, respectively. The other conditions remained consistent with those used in the energy resolution study. The simulated peak-to-total ratios for different energy γ-rays were obtained as shown in Table 3 and were compared with the experimental peak-to-total ratio values from the literature. In the literature, the experimental peak-to-total ratio was measured using a NaI(Tl) detector with a crystal size of Φ3 inch × 3 inch, and its crystal dimensions, encapsulation, and other main structures are consistent with those in this simulation study.

**Figure 9:**
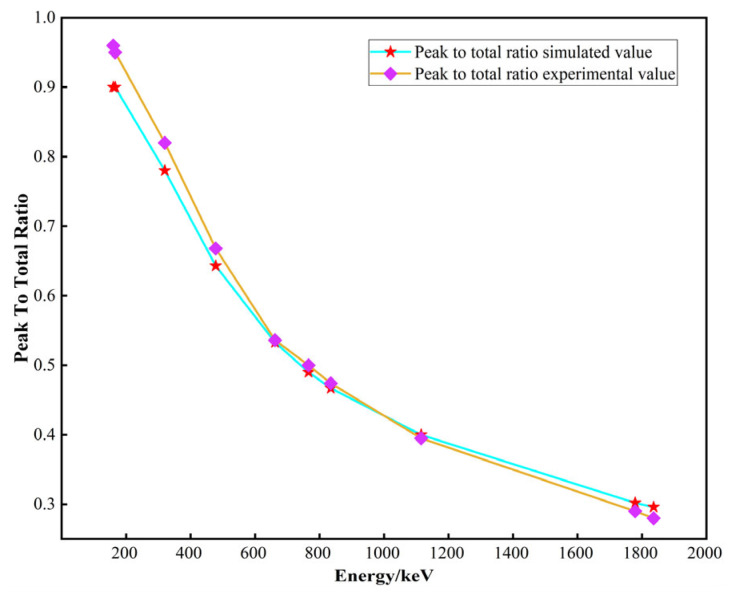
Variation trend of detector peak-to-total ratio with incident gamma-ray energy.

As shown in Table 3, the error between the simulated and experimental values of the peak-to-total ratio is within 6.25%, verifying the accuracy of the simulation data. The relationship curve between the detector’s peak-to-total ratio and the incident ray energy variation is plotted in Figure 9.

As can be seen from Figure 9, the simulated and experimental values of the peak-to-total ratio show good agreement in their variation trends, both decreasing with increasing incident ray energy. This is primarily because, as the incident ray energy increases, the photoelectric effect cross section decreases while the Compton effect cross section increases. Additionally, the probability of ray particles penetrating the crystal and escaping into the sensitive region of the detector becomes higher, leading to a reduction in the full-energy peak counts of the detector and consequently a decrease in the peak-to-total ratio. Therefore, NaI detectors exhibit better spectral detection efficiency for low-energy γ-rays.

## 4. Conclusions

This study investigated the effects of the crystal size, ray energy, cladding thickness, and detection distance on the absolute detection efficiency, full-energy peak detection efficiency, and intrinsic detection efficiency of NaI(Tl) detectors using the TopMC tool based on the Monte Carlo method. Furthermore, simulations were conducted to calculate the energy resolution and peak-to-total ratio of the detector. The study found the following: (1) All three detection efficiencies showed a positive correlation with crystal size. (2) As the radiation energy increased, all three detection efficiencies first rose and then declined, reaching their maximum values at 100 keV: 8.63%, 7.72%, and 92.2%, respectively. (3) An increase in the thickness of the aluminum cladding had almost no effect on the intrinsic detection efficiency but led to a reduction in both the absolute detection efficiency and the full-energy peak detection efficiency. (4) With increasing detection distance, the absolute detection efficiency and full-energy peak detection efficiency decreased, while the intrinsic detection efficiency first decreased and then increased, reaching its lowest value of 51.2% at a detection distance of 2.5 cm. (5) The energy resolution improved as the radiation energy increased. The simulated energy resolution values for the ^137^Cs and ^60^Co sources were close to the experimental values, but the resolution for the low-energy γ-rays emitted by the ^241^Am source was relatively poor. (6) The simulated peak-to-total ratios for different radiation sources showed errors within 6.25% compared to the experimental values, and the simulated peak-to-total ratio decreased with increasing incident photon energy, which is consistent with the trend of the experimental values as a function of energy. In summary, NaI(Tl) detectors exhibit better detection efficiency when measuring low-energy gamma-ray spectra. When designing NaI(Tl) detectors for low-energy response, the optimal detection efficiency can be achieved by considering larger crystal dimensions while selecting the thinnest possible end-cap housing thickness that maintains mechanical strength. These research findings enhance our understanding of the influencing factors and relevant principles governing the detection performance of NaI(Tl) detectors. The results provide valuable guidance for detector design, passive efficiency calibration, and practical applications, contributing to the conservation of production and time costs.

## Figures and Tables

**Table 1:** Detection efficiency for different crystal sizes.

Detect Crystal Type	Crystal Size/Inch × Inch *	*ε_abe_* (%)	*ε_p_* (%)	*ε_in_* (%)
NaI(Tl)	Φ1 × 1	0.52	0.15	37.1
	Φ2 × 2	2.39	1.03	47.8
	Φ3 × 3	5.19	2.71	54

* 1 inch = 2.54 cm.

**Table 2:** Simulated and experimental energy resolution values for γ-rays with different incident energies.

Source	Energy/keV	Energy Resolution			
		Simulated Value		Experimental Value [16]	
		*FWHM*/keV	*R*/%	*FWHM*/keV	*R*/%
^241^Am	59.5	15.9	26.7	9.57	16.1
^137^Cs	662	46	6.95	47.53	7.2
^60^Co	1332.5	79	5.93	66.87	5

**Table 3:** List of simulated peak-to-total ratios *ε_f_* and experimental measurements for γ-rays with different incident energies.

Nuclide	γ-Ray Energy/keV	Peak-to-Total Ratio *ε_f_*		
		Simulated Value	Experimental Value [17]	Error/%
^47^Sc	160	0.900	0.960	6.25
^139^Ce	166	0.900	0.950	5.26
^51^Cr	320	0.780	0.820	4.88
^7^Be	478	0.643	0.668	3.74
^137^Cs	662	0.533	0.536	0.56
^95^Nb	766	0.490	0.500	2.00
^54^Mn	835	0.467	0.474	1.48
^65^Zn	1115	0.400	0.395	1.27
^28^Al	1779	0.302	0.290	4.14
^88^Y	1836	0.296	0.280	5.71

## Data Availability

The data generated during the experimental process were used for detector performance prediction and structural optimization research. Due to project confidentiality requirements, the raw data cannot be publicly shared. This paper presents the complete aggregated experimental results related to the study of detector performance.
